# PAI: Predicting adenosine to inosine editing sites by using pseudo nucleotide compositions

**DOI:** 10.1038/srep35123

**Published:** 2016-10-11

**Authors:** Wei Chen, Pengmian Feng, Hui Ding, Hao Lin

**Affiliations:** 1 Department of Physics, School of Sciences, and Center for Genomics and Computational Biology, North China University of Science and Technology, Tangshan, 063000, China; 2 School of Public Health, North China University of Science and Technology, Tangshan, 063000, China; 3 Key Laboratory for Neuro-Information of Ministry of Education, School of Life Science and Technology, Center for Informational Biology, University of Electronic Science and Technology of China, Chengdu 610054, China

## Abstract

The adenosine to inosine (A-to-I) editing is the most prevalent kind of RNA editing and involves in many biological processes. Accurate identification of A-to-I editing site is invaluable for better understanding its biological functions. Due to the limitations of experimental methods, in the present study, a support vector machine based-model, called **PAI,** is proposed to identify A-to-I editing site in *D. melanogaster*. In this model, RNA sequences are encoded by “pseudo dinucleotide composition” into which six RNA physiochemical properties were incorporated. **PAI** achieves promising performances in jackknife test and independent dataset test, indicating that it holds very high potential to become a useful tool for identifying A-to-I editing site. For the convenience of experimental scientists, a web-server was constructed for **PAI** and it is freely accessible at http://lin.uestc.edu.cn/server/PAI.

The adenosine to inosine (A-to-I) editing is the most prevalent kind of RNA editing, which has been found from fungi to human^1^. A-to-I editing is catalyzed by the highly conserved enzyme ADARs (adenosine deaminases that act on RNA), which bind dsRNA (double-stranded RNA) structures and deaminate the targeted A within these structures into I^2^,^3^, Fig. 1.

The inosine yielded by A-to-I editing replaces the genomically encoded adenosine and is read by the cellular machinery as a guanosine (G)^3^,^4^,^5^. Therefore, A-to-I editing not only results in codon changes^6^, but also serves numerous post-transcriptional roles, such as regulating alternative splicing, modifying microRNA gene products and altering their microRNA target sites^7^,^8^,^9^. Therefore, the knowledge about the positions of A-to-I editing site is important for deciphering its biological functions.

By using RNA-Seq method, A-to-I editing sites have been detected in *H. sapiens*^10^,^11^,^12^, *M. musculus*^13^, and *D. melanogaster*^14^. The experimental data yielded quite encouraging results and play a role in promoting the research progress on identifying the distribution of A-to-I editing site. However, the high error rates of many next-generation sequencing platforms present a major challenge for A-to-I editing site discovery^14^. Therefore, it is in high demand to develop computational methods for analyzing the distribution and function of A-to-I editing site, so as to speed up genome-wide A-to-I editing site detection.

Based on the RNA-Seq data, Laurent and his colleague constructed a high quality dataset and developed a computational model to detect A-to-I editing site in *D. melanogaster*^14^. However, the features used in their model are all information from RNA-Seq experiment. Therefore, their model couldn’t be used to detect A-to-I editing site in the cases without the reads information from RNA-Seq experiment. Moreover, no web-server or predictor was provided for their method, and hence its usage is quite limited, particularly for the broad experimental scientists.

Keeping this in mind, in the present study, we proposed a support vector machine (SVM) based-method to identify the A-to-I editing site in *D. melanogaster*. By using the pseudo dinucleotide composition as the input feature vector of support vector machine, the long-range sequence-order effects and RNA physicochemical properties were integrated together in the proposed model. It is encouraging that the proposed method obtained promising performances in jackknife test and independent dataset test. For the convenience of experimental scientists, a web-server for the proposed model is provided at http://lin.uestc.edu.cn/server/PAI.

## Result and Discussion

### Parameter optimization

By using PseDNC, RNA samples in the benchmark dataset can be transferred to a discrete vector whose dimension and elements depend on the two parameters *w* and *λ* (see Materials and Methods). *w* is the weight factor usually within the range from 0 to 1, and *λ* is the global order effect. Generally speaking, the greater the *λ* is, the more global sequence-order information the model contains. However, if *λ* is too large, it would reduce the cluster-tolerant capacity so as to lower down the cross-validation accuracy due to over-fitting or “high dimension disaster” problem. Therefore, our searching for the optimal values of the two parameters is in the range of *w*∈[0, 1] and *λ*∈[1, 10] with the steps of 0.1 and 1, respectively.

**Figure 2:**
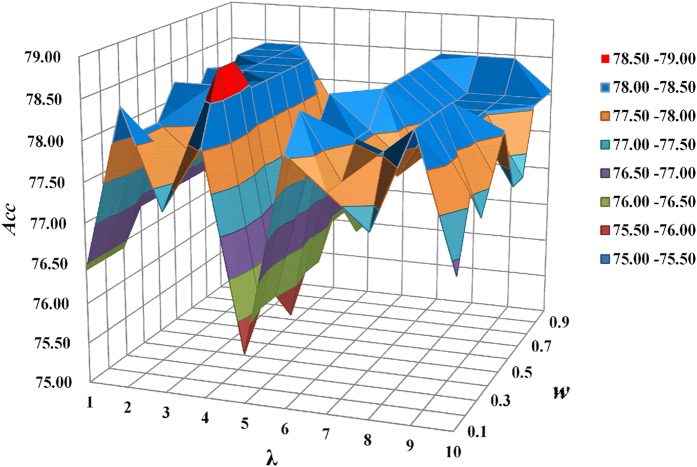
A graph to show the accuracies obtained in the 5-fold cross-validation with different values of w and λ.

In order to reduce the computational time, the 5-fold cross-validation method was used to optimize the two parameters. We found that when *w* = 0.3 and *λ* = 4, a peak of 78.86% was obtained for the *Acc* (Fig. 2). Accordingly, these two numerical values, *w* = 0.3 and *λ* = 4, were used for the two uncertain parameters to build the SVM-based model. The model thus obtained is called **PAI**, where “P” stands for Predicting, “A” for Adenosine and “I” for Inosine.

### A-to-I editing site sites prediction

The jackknife test is the least arbitrary and most objective cross-validation method and has been increasingly adopted by researchers to examine the quality of various computational models. Thus, the jackknife test was used to examine the performance of **PAI**. In the jackknife test, **PAI** obtained an accuracy of 79.51% with the sensitivity of 85.60%, specificity of 73.11% and MCC of 0.60 for identifying A-to-I editing sites in the benchmark dataset. To further testify its performance, we also applied the **PAI** to identify the 300 A-to-I editing sites in the independent dataset, and found that **PAI** could correctly identify 247 A-to-I editing sites with the sensitivity of 82.33%.

### Comparison with other classifiers

Since there is no freely accessible predictor or webserver that could be used to identify the A-to-I editing sites, and hence no comparison could be made in this study for **PAI** with its counterparts. In order to testify its superiority, we compared the predictive results of **PAI** with those of other commonly used classifiers, i.e., Naïve Bayes, BayesNet and J48 Tree, as implemented in WEKA^15^. The jackknife test results of different classifiers for identifying A-to-I editing sites in the benchmark dataset were reported in Table 1.

It is shown that the sensitivity, specificity, accuracy and MCC of **PAI** are all higher than that of the other three state-of-the-art classifiers. These results suggest that the proposed SVM based model can be effectively used to identify A-to-I editing sites.

### Webserver

To enable applications of the proposed method and for the convenience of scientific community, a freely accessible online webserver was established. The user guide is given as following.

#### Step 1

**Figure 3:**
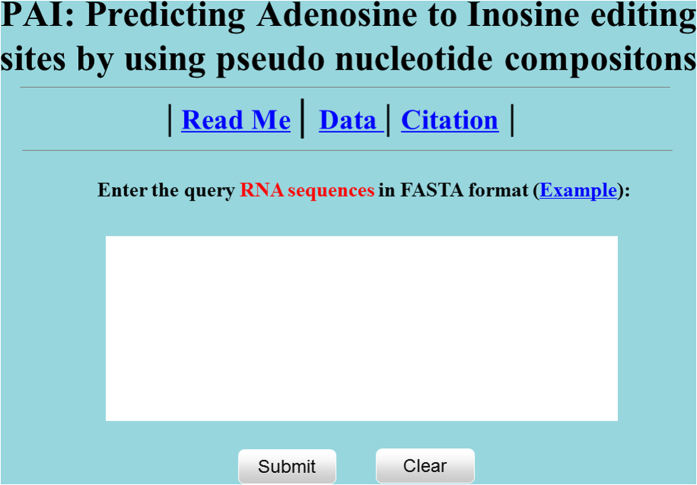
A semi-screenshot for the top-page of the PAI web-server at http://lin.uestc.edu.cn/server/PAI.

Open the web server at http://lin.uestc.edu.cn/server/PAI, and the top page of **PAI** will be shown as in Fig. 3.

#### Step 2

Either type or copy/paste the query RNA sequences into the input box at the center of Fig. 3.

#### Step 3

Click on the ‘Submit’ button to see the predicted result. For example, if use the query RNA sequences in the ‘Example’ window as the input, the outcomes are as following: All the Adenosines (A) at position 26 in the four query sequences can be edited to Inosine (I). These results are consistent with the experimental observations.

## Conclusions

RNA-seq analyses have demonstrated that A-to-I editing is associated with a number of key biological processes and plays important roles ranging from changing codon to regulating mRNA splicing. Therefore, genome-wide detection of A-to-I editing sites will facilitate our understanding of its biological functions.

In the present study, we proposed a support vector machine based model for predicting A-to-I editing sites by using pseudo dinucleotide composition and found that the model is very promising as reflected by high success rates obtained from the rigorous jackknife test and independent dataset test.

For the convenience of researchers in the scientific community, a web-server for the proposed model, called **PAI**, is provided. We hope that it will provide novel insights into the understanding of the distribution and function of A-to-I editing. As the current method is only applicable to *D. melanogaster*, future work will expand to other species once the high quality experimental data that can be used to train the model is available.

## Materials and Methods

### Dataset

The benchmark dataset used to train and test the proposed method was built based on Laurent *et al*.’s work^14^. By using single molecular sequencing method, they sequenced the RNAs and DNAs of the wild-type *D. melanogaster* and RNAs of the ADAR-deficient *D. melanogaster*, and obtained a training dataset including 127 A-to-I editing site containing sequences and 127 non-A-to-I editing site containing sequences. After removing the redundant samples in their dataset, we obtained a benchmark dataset including 125 A-to-I editing site containing sequences and 119 non-A-to-I editing site containing sequences.

It was observed via preliminary trials that when the length of the sequences in the benchmark dataset is 51 nt with the A that can be edited to Inosine in the center, the corresponding predictive results were most promising. Accordingly, all the sequences in the training dataset are 51-nt long and are available at http://lin.uestc.edu.cn/server/PAI.

To further verify the power of the proposed method, we also build an independent dataset by harvesting the A-to-I editing site containing sequences of *D. melanogaster* from Yu and his colleagues’ work^16^. By removing the sequences with more than 75% sequence similarity using CD-HIT^17^, we obtained 300 A-to-I editing site containing sequences. These sequences are also 51-nt long and are available at http://lin.uestc.edu.cn/server/PAI.

### Pseudo nucleotide composition

In order to include the global sequence order information, the pseudo nucleotide composition was proposed to represent genomic sequences^18^. Since its introduction, pseudo nucleotide composition has been successfully applied in many branches of computational genomics^19^,^20^,^21^,^22^. Due to its excellent performance, a series of flexible web-servers were developed to generate pseudo nucleotide compositions^23^,^24^,^25^,^26^. Therefore, in the current work, the pseudo nucleotide composition was also used to represent RNA samples. Below is the brief elaboration on how to encode RNA sequences using pseudo nucleotide composition. For more details of pseudo nucleotide composition, see a recent review^27^.

Suppose a RNA sequence with *L* nucleic acid residues, the pseudo nucleotide composition can be defined as,


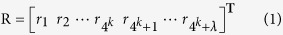


where


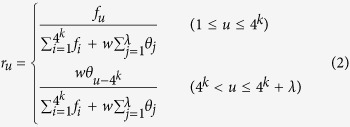


In Eq. 2, *f*_*u*_ (*u* = 1, 2, …., 4^*k*^) is the normalized occurrence frequency of the non-overlapping *k*-tuple nucleotides in the RNA sequence. *λ* is the number of the total counted ranks of the correlations along a RNA sequence, and *w* is the *w*eight factor. It is through the *λ* correlation factors that not only considerable global sequence-order effects can be incorporated but the RNA sequences in the benchmark dataset with extreme difference in length can also be converted into a set of feature vectors with a same dimension. The correlation factor *θ*_*j*_ represents the *j*-tier structural correlation factor between all the *j*-th most contiguous *k*-tuple nucleotide T_*i*_ = R_*i*_R_*i+*1_…R_*i+k*−1_ and is defined as,


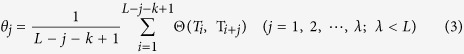


For example, *θ*_1_ is called the first-tier correlation factor that reflects the sequence order correlation between all the most contiguous *k*-tuple nucleotide along a RNA sequence; *θ*_2_, the second-tier correlation factor between all the second most contiguous *k*-tuple nucleotide; *θ*_3_, the third-tier correlation factor between all the third most contiguous *k*-tuple nucleotide; and so forth. The correlation function Θ(T_*i*_, T_*j*_) is given by


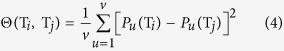


where *v* is the number of RNA physicochemical properties. *P*_*u*_(T_*i*_) is the numerical value of the *u*-th (*u* = 1, 2, …., 6) property for the dinucleotide T_*i*_at position *i*, and *P*_*u*_(T_*j*_) is the corresponding value for the dinucleotide T_*j*_ at position *j*.

Before substituting them into Eq. 4, all the original values *P*_*u*_(T_*i*_) (*u* = 1, 2, …, 6) were subjected to a standard conversion as described by the following equation,


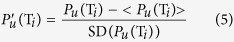


where the symbol <> means taking the average of the quantity therein over the 16 different dinucleotides, and SD means the corresponding standard deviation. The converted values obtained by Eq. 5 will have a zero mean value over the 16 different dinucleotides.

### RNA physicochemical properties

It has been reported that A-to-I editing are correlated with RNA structures^2^. Since RNA structure is determined by the complex pattern of base-base interaction^28^,^29^,^30^,^31^, the RNA local structural properties were used to define the pseudo nucleotide composition, of which three are local translational parameters (Shift, Slide, Rise) and the other three the local angular parameters (Twist, Tilt, Roll). The detailed values for the six local structural property parameters are given in Table 2. Therefore, *k* is equal to 2 meaning that the pseudo dinucleotide composition (PseDNC) was used, and *v* is equal to 6 reflecting the number of RNA physicochemical properties considered.

### Support Vector Machine

As a smart supervised machine learning algorithm, support vector machine (SVM) has been widely employed to build classifiers in the realm of computational genomics and proteomics^32^,^33^,^34^,^35^,^36^. Its basic idea is to transform the input data into a high dimensional feature space and then determine the optimal separating hyperplane. In the current study, the LibSVM package 3.18 (https://www.csie.ntu.edu.tw/~cjlin/libsvm/) was used to perform the predictions. The radial basis function (RBF) was chosen as the kernel of SVM, where the regularization parameter *C* and kernel parameter *γ* were optimized using a grid search approach as defined by


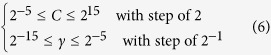


### Performance evaluation

The performance of the proposed method was evaluated by using the widely used four metrics, namely sensitivity (*Sn*), specificity (*Sp*), Accuracy (*Acc*) and the Mathew’s correlation coefficient (*MCC*), which are expressed as


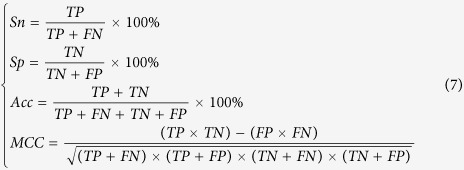


where TP represents the number of the correctly recognized A-to-I editing site containing sequences, TN represents the number of the correctly recognized non-A-to-I editing site containing sequences, FP represents the number of non-A-to-I editing site containing sequences recognized as A-to-I editing site containing sequences and FN represents the number of A-to-I editing site containing sequences recognized as non-A-to-I editing site containing sequences, respectively.

## Additional Information

**How to cite this article**: Chen, W. *et al*. PAI: Predicting adenosine to inosine editing sites by using pseudo nucleotide compositions. *Sci. Rep*. **6**, 35123; doi: 10.1038/srep35123 (2016).

## Figures and Tables

**Figure 1:**
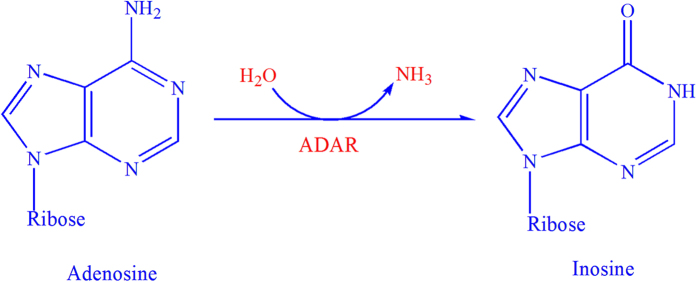
Illustration to show the adenosine to inosine. Its formation is catalyzed by the enzyme ADARs (adenosine deaminases that act on RNA).

**Table 1:** Comparison of different methods for identifying A-to-I editing site by the jackknife test.

Method	Sn (%)	Sp (%)	Acc (%)	MCC
Naïve Bayes	81.60	71.40	76.60	0.53
BayseNet	81.60	69.70	75.80	0.52
J48	67.50	63.10	65.40	0.31
PAI	85.60	73.11	79.51	0.60

**Table 2:** The original values for the six RNA dinucleotide physical structures.

Dinucleotide	Shift (nm)	Slide (nm)	Rise (nm)	Tilt (°)	Roll (°)	Twist (°)
AA	−0.08	−1.27	3.18	−0.80	7.00	31.00
AC	0.23	−1.43	3.24	0.80	4.80	32.00
AG	−0.04	−1.50	3.30	0.50	8.50	30.00
AU	−0.06	−1.36	3.24	1.10	7.10	33.00
CA	0.11	−1.46	3.09	1.00	9.90	31.00
CC	−0.01	−1.78	3.32	0.30	8.70	32.00
CG	0.30	−1.89	3.30	−0.10	12.10	27.00
CU	−0.04	−1.50	3.30	0.50	8.50	30.00
GA	0.07	−1.70	3.38	1.30	9.40	32.00
GC	0.07	−1.39	3.22	0.00	6.10	35.00
GG	−0.01	−1.78	3.32	0.30	12.10	32.00
GU	0.23	−1.43	3.24	0.80	4.80	32.00
UA	−0.02	−1.45	3.26	−0.20	10.70	32.00
UC	0.07	−1.70	3.38	1.30	9.40	32.00
UG	0.11	−1.46	3.09	1.00	9.90	31.00
UU	−0.08	−1.27	3.18	−0.80	7.00	31.00
